# Attractive Interactions among Intermediate Filaments Determine Network Mechanics In Vitro

**DOI:** 10.1371/journal.pone.0093194

**Published:** 2014-04-01

**Authors:** Paul Pawelzyk, Norbert Mücke, Harald Herrmann, Norbert Willenbacher

**Affiliations:** 1 Institute for Mechanical Process Engineering and Mechanics, Karlsruhe Institute of Technology (KIT), Karlsruhe, Germany; 2 Biophysics of Macromolecules, German Cancer Research Center, Heidelberg, Germany; 3 Division of Molecular Genetics, German Cancer Research Center, Heidelberg, Germany; Dalhousie University, Canada

## Abstract

Mechanical and structural properties of K8/K18 and vimentin intermediate filament (IF) networks have been investigated using bulk mechanical rheometry and optical microrheology including diffusing wave spectroscopy and multiple particle tracking. A high elastic modulus *G*_0_ at low protein concentration *c*, a weak concentration dependency of *G*_0_ (*G*_0_∼*c* ^0.5±0.1^) and pronounced strain stiffening are found for these systems even without external crossbridgers. Strong attractive interactions among filaments are required to maintain these characteristic mechanical features, which have also been reported for various other IF networks. Filament assembly, the persistence length of the filaments and the network mesh size remain essentially unaffected when a nonionic surfactant is added, but strain stiffening is completely suppressed, *G*_0_ drops by orders of magnitude and exhibits a scaling *G*_0_∼*c* ^1.9±0.2^ in agreement with microrheological measurements and as expected for entangled networks of semi-flexible polymers. Tailless K8Δ/K18ΔT and various other tailless filament networks do not exhibit strain stiffening, but still show high *G*_0_ values. Therefore, two binding sites are proposed to exist in IF networks. A weaker one mediated by hydrophobic amino acid clusters in the central rod prevents stretched filaments between adjacent cross-links from thermal equilibration and thus provides the high *G*_0_ values. Another strong one facilitating strain stiffening is located in the tail domain with its high fraction of hydrophobic amino acid sequences. Strain stiffening is less pronounced for vimentin than for K8/K18 due to electrostatic repulsion forces partly compensating the strong attraction at filament contact points.

## Introduction

Mechanical properties of metazoan cells are determined by three distinct types of filament systems: F-actin, intermediate filaments (IFs) and microtubules [1]. IFs are by far the most diversified cytoskeletal filaments in humans, encoded by 70 genes [2]. A common feature of IFs is the basic building block consisting of dimeric complexes with a central α-helical rod domain and a non-helical head and tail. These complexes assemble into filaments with a diameter of 10 nm and a persistence length *l*_p_ on the order of 0.3–1 µm [3], [4]. Common features of pure IF networks at physiological conditions are the pronounced elasticity at small deformations as characterized by a frequency independent storage modulus *G*_0_ and the weak dependence of *G*_0_ on protein concentration found for vimentin [5]–[7], desmin [7] and keratin [6]. This property strongly disagrees with the results obtained for actin networks [5], [8] and contradict existing theoretical models for networks of flexible or semi-flexible polymers [8]–[11]. Only at protein concentrations above 1.5 g/l or at divalent ions concentrations above 2 mM, *G*_0_ and its scaling with protein concentration are similar to what is expected from the above mentioned theoretical models [12], [13].

Yamada et al. [14] showed that the modulus of K8/K18 networks drastically decreases when phospholipids or the non-ionic surfactant Triton X-100 (TX-100) are added, but that this has no effect on the network structure as revealed by electron microscopy and on the polymerization state of the protein as determined by pelleting experiments. These authors also hypothesized, that the high shear moduli found for K8/K18 networks may be inferred from the elasticity of the air/liquid interface. However, we showed that artifacts from surface elasticity, sample preparation, or wall slip on the linear viscoelastic properties of K8/K18 networks do not cause the unusual linear viscoelastic properties [15]. Furthermore, we have demonstrated that there must be an additional contribution to the free energy of the network resulting in the high *G*_0_ values and their weak dependence on protein concentration [15]. Following the theory for swollen networks of cross-linked polymers [16], we suggested that this contribution originates from a stretched conformation of filaments between cross-links.

Strain stiffening of IF networks, i.e. the pronounced increase of the elastic modulus at high stresses or strains is a hallmark feature of IF networks, which is of special physiological relevance since strain stiffening is reduced for IF mutations related to the blistering disease epidermolysis bullosa simplex [17] or severe skeletal and cardiac myopathies [18]. For actin filament networks, strain stiffening is closely related to cross-links of filaments induced by the introduction of external cross-linking proteins [19], [20]. Strain stiffening of uncross-linked F-actin solutions was explained by unspecific attractions controlled by temperature, ionic strength, filament length and protein concentration [21]. Networks of various IFs also exhibit strain stiffening even in the absence of external cross-links like divalent cations, plectin or desmoplakin [7], [12]. The attractive interactions among IFs responsible for strain stiffening seem to be more specific. For several systems like vimentin, neurofilaments and K8/K18 stiffening is observed in a wide range of protein and salt concentrations [12], [13], [15].

Strong attractive interactions among filaments at their contact points are required to maintain a network of stretched filaments as well as strain stiffening, but the control of these attractive interactions and the responsible sequence motifs within the protein remain elusive.

## Results

Mesh size and homogeneity of K8/K18 filament networks are directly obtained from multiple particle tracking (MPT) experiments. The persistence length of filaments is determined from high frequency mechanical squeeze flow as well as diffusing wave spectroscopy (DWS) microrheology. The linear and non-linear viscoelastic network properties are characterized employing classical shear rheometry. In particular, we will discuss the effect of the non-ionic surfactant TX-100 on these network properties. Results for K8/K18 will be compared to those obtained for IF networks assembled from tailless K8 and tailless K18 (K8Δ/K18ΔT) and from the mesenchymal IF protein vimentin.

### Mesh size of K8/K18 networks

**Figure 1:**
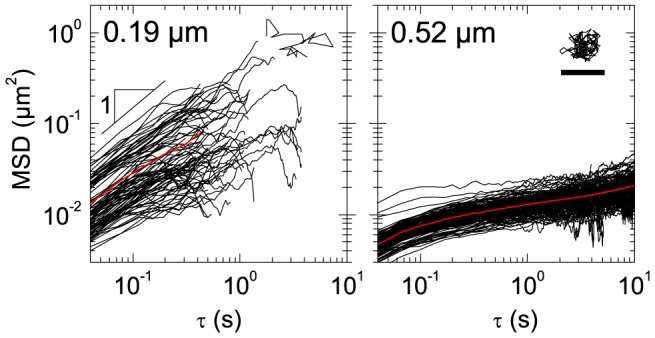
Determination of the mesh size by multiple particle tracking. MSDs against time *τ* and the individual trajectories after *τ* = 3 s (inset) of microspheres with diameters of 0.19 and 0.52 µm at a K8/K18 concentration of 1.0 g/l (19.7 µM). The scale bar for the trajectory shown in the right panel denotes 500 nm and is the same in both panels and directions. The red line represents the average MSD.

To determine the mesh size *ξ* by MPT, we assembled K8/K18 networks in the presence of well-dispersed tracer particles and monitored the thermal motion of these fluorescent particles using video microscopy. The mean square displacement (MSD) is a measure of the average distance a particle travels within a given time interval *τ*. Figure 1 shows the MSD of randomly chosen particles with a diameter of 0.19 µm and 0.52 µm at a K8/K18 concentration of 1.0 g/l. The particles with a diameter of 0.19 µm diffuse freely through the network and the MSD increases linearly with time as expected for a viscous fluid. In contrast, diffusion of the 0.52 µm particles is confined by the network and the MSD approaches a constant value at long times *τ*. Accordingly, the mesh size determined by MPT is between 0.19 µm and 0.52 µm. The mesh size *ξ* can also be estimated from protein concentration *c* assuming a cubic network of rigid rods:
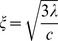
with the mass per unit length *λ* = 3.16·10^−11^ g/m for K8/K18 [22]. For *c* = 1.0 g/l, this results in *ξ* = 0.31 µm, which agrees well with the results from MPT. This simple estimate is a good approximation for semi-flexible wormlike chains if *l*_p_≈*ξ*. Good agreement between the mesh size from MPT and the cubic model has also been reported for vimentin networks [23].

### Persistence length of K8/K18 filaments

**Figure 2:**
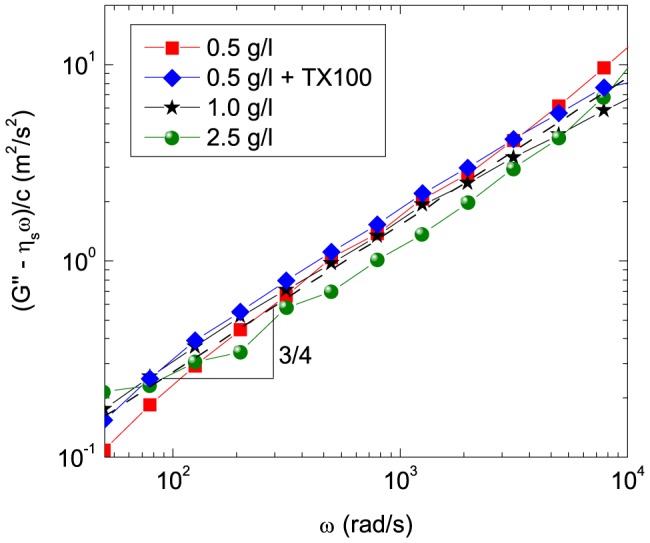
Determination of the persistence length from high frequency oscillatory squeeze flow measurements. The reduced linear viscoelastic loss modulus *G″-ωη*_s_ measured by squeeze flow normalized by concentration as a function of frequency *ω*. The dashed line represents a fit with a slope of ¾, which results in of *l*_p_ = 0.65±0.1 µm using equation (2).

In the high frequency limit, the viscoelastic properties originate from bending fluctuations of individual filament segments. In this regime, the modulus of semi-flexible polymers scales with angular frequency *ω* as G*∼*ω* ^3/4^ [24]. These bending fluctuations are related to the persistence length *l*_p_ or the bending modulus *κ* = *k*_B_ *Tl*_p_ with the Boltzmann constant *k*_B_ and the temperature *T*. At high frequencies, these quantities are related to the complex modulus G*(*ω*) [24]:
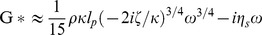
with the buffer viscosity *η*_s_ = 1 mPa s, and the drag coefficient *ζ*. According to [25], the drag coefficient is given by *ζ*≈2*πη*_s_/ln(*ξ*/*d*) with the filament diameter *d* and the mesh size *ξ*, which is calculated from *λ* and *c* according to equation (1). The piezo-driven oscillatory squeeze flow rheometer is a mechanical method to determine the viscoelastic properties in the frequency range between *ω* = 10 rad/s and 3·10^4^ rad/s. Figure 2 shows that the measured data of the viscous modulus *G*″–*ωη*_s_ depends linearly on concentration and scales with *ω* ^3/4^. Thus, the persistence length *l*_p_ can be calculated according to equation (2). We obtain a persistence length of *l*_p_ = 0.65±0.1 µm, which is in the range of the value of 0.3 µm determined from the analysis of the curvature of K8/K18 filaments imaged by microscopic methods [3]. Similar values were found for desmin and vimentin [7]. The persistence length *l*_p_ is in the range of the mesh size *ξ* in the concentration range investigated here. Hence, the mesh size can be estimated according to equation (1) assuming a cubic grid of rigid filaments.

### Effect of TX-100 on structure and linear viscoelasticity

**Figure 3:**
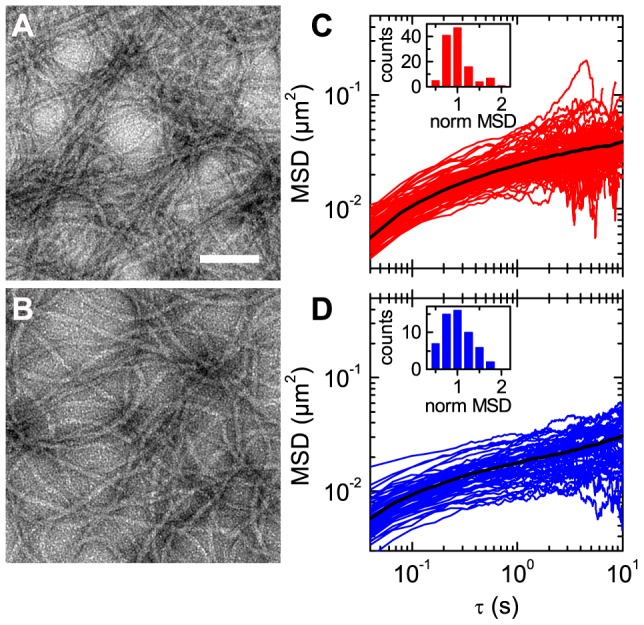
Influence of TX-100 on network structure of K8/K18 characterized by electron microscopy and MPT. Electron micrographs of K8/K18 without surfactant (A) and with 0.01% TX-100 (B). Scale bar represents 100 nm. (C) and (D) show the MSDs without surfactant and with 0.01% TX-100 at *c* = 1.0 g/l (19.7 µM) as a function of lag time *τ*. The black line illustrates the ensemble average of the MSDs. The insets show the histogram of the MSDs normalized by the averaged MSD after *τ* = 1 s.

Transmission electron micrographs of negatively stained K8/K18 filaments without surfactant (Figure 3A) and in the presence of 0.01% TX-100 (Figure 3B) show homogeneous long filaments. The K8/K18 network without surfactant seems to look more dense, likely because of slight differences of the filament deposition on the grid as discussed in ref. [22], but in general, these images do not reveal qualitative differences in filament assembly or network structure due to the addition of surfactant.

MPT microrheology was also used to probe the influence of surfactant on network microstructure in its natural aqueous environment. Particles (*d* = 0.52 µm) slightly bigger than the mesh size at the respective protein concentration *c* = 1.0 g/l were used and corresponding MSD data for networks without surfactant and in the presence of TX-100 are presented in Figure 3C and D. The MSD data for both cases follow the same trend and have approximately the same magnitude. At long times *τ* the MSD approaches a time independent plateau showing that the particles are trapped within the network. The insets show the histogram of the MSDs at *τ* = 1 s. Not only the average MSD, but also the distribution of MSDs is very similar for both networks and the addition of TX-100 does not show any significant effect. Similar results were observed for homogeneous F-actin solutions [26].

The influence of surfactant TX-100 on the persistence length *l*_p_ of individual filaments was determined using high frequency squeeze flow rheology (Figure 2). For K8/K18, *l*_p_ is unaffected by the surfactant.

**Figure 4:**
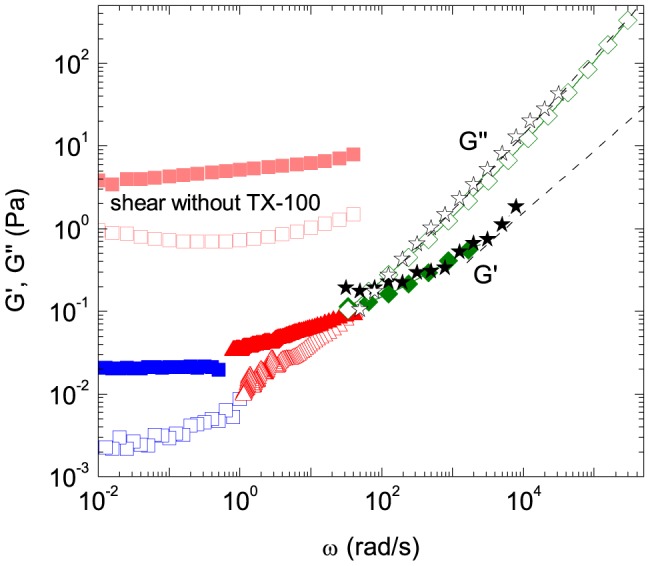
Frequency dependence of the linear viscoelastic moduli *G′* and *G″* of K8/K18. *G′* (closed symbols) and *G″* (open symbols) obtained from shear rheology (squares), MPT (red triangles), and DWS (green diamonds) and squeeze flow (black stars) at a concentration of 0.5 g/l (9.8 µM). Dashed lines: Theoretical values calculated using equation (2) with a persistence length of *l*_p_ = 0.65 µm and the mesh size according to equation (1).

The frequency dependence of the storage modulus *G′* and the loss modulus *G″* of a K8/K18 network is shown in Figure 4. Data at low frequencies were obtained from MPT and shear rheometry. The latter experiments were performed in the linear viscoelastic regime below the critical strain 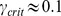 at which the viscoelastic response becomes non-linear. These methods cover the frequency range up to 100 rad/s. Oscillatory squeeze flow and DWS have been used to expand the frequency range beyond 10^5^ rad/s. DWS is a microrheological method that measures the temporal fluctuations of the light scattered by tracer particles. The average MSD of the particles determined from the intensity auto-correlation function was used to calculate *G′* and *G″* of the surrounding fluid [27]. In an analogous manner, *G′* and G″ data were obtained from MPT result [28]. Good agreement between G* data from bulk rheometry and DWS has been confirmed in a wide frequency range for polymer and surfactant solutions [29]. This method is applied here to IF networks for the first time. K8/K18 networks with and without surfactant exhibit both a predominantly elastic gel-like behavior in the frequency range accessible by shear rheometry, but the moduli drop by two orders of magnitude upon addition of a critical concentration of TX-100. This effect of surfactant on the results of oscillatory shear measurements has been reported previously [14]. In contrast, squeeze flow, DWS and MPT data are not affected by the surfactant (Figure 2 and Figure S1) and agree well with shear rheological data obtained in the presence of TX-100 (Figure 4). This is the first time that the linear viscoelastic properties of an IF network have been determined over such a broad frequency range using four independent methods.

**Figure 5:**
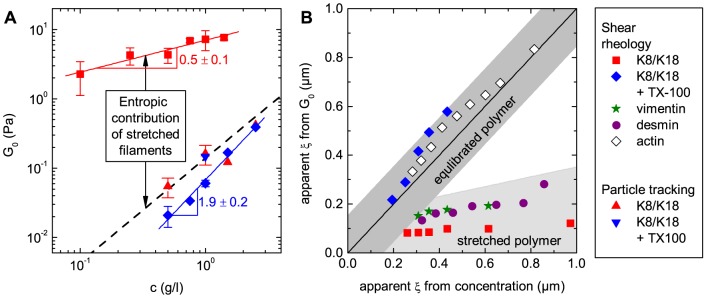
Influence of protein concentration on *G*_0_ and comparison of the respective apparent mesh sizes *ξ*. (A) presents *G*_0_ data obtained from shear rheology and particle tracking at *ω* = 1 rad/s as a function of K8/K18 concentration. The dotted line shows the results obtained by equation (4). (B) Comparison of the mesh sizes *ξ* for different biological filament networks calculated from protein concentration using the cubic grid model in equation (1) with the mesh size calculated from the plateau modulus *G*_0_ according to equation (3). The data used for K8/K18 is the same as in (A). Data for vimentin and desmin were taken from [7]. Actin data was extracted from Fig. 3 in [8]. The dark grey zone illustrates the region where the simplistic model can be used to calculate the mesh size of networks from G_0_ with an uncertainty of ±0.15 µm. This holds if the filaments are in thermal equilibrium. The light grey area shows that this is not the case for many IF-networks.

We have characterized the linear viscoelastic properties of K8/K18 networks with and without surfactant at different protein concentrations. Corresponding data of the frequency independent elastic modulus *G′* also termed plateau modulus *G*_0_ are shown in Figure 5A. In addition to shear rheological data, we plot *G*_0_ determined from MPT measurements. In the presence of TX-100, the absolute values of *G*_0_ decrease drastically especially at low concentrations and the scaling exponent α characterizing the concentration dependence of *G*_0_∼*c^α^* increases from *α* = 0.5±0.1 to 1.9±0.2. Most strikingly, the data from MPT experiments without surfactant are close to the results from mechanical shear rheometry with TX-100.

Assuming thermal equilibrium, where only chemical or physical constraints at contact points contribute to *G*_0_, the mesh size *ξ* can be estimated:
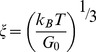

Figure 5B compares *ξ* of K8/K18 and literature data for vimentin, desmin and actin calculated from the protein concentration using equation (1) with the apparent *ξ* calculated from *G*_0_ according to equation (3). The length densities of the filaments used in Figure 5B are *λ* = 6.31·10^−11^ g/m for vimentin [22], *λ* = 9.80·10^−11^ g/m for desmin [22] and *λ* = 2.66·10^−11^ g/m [30] for actin. The data calculated for networks of K8/K18 filaments with surfactant and actin filaments are close to the diagonal line, showing that both methods result essentially in the same mesh size *ξ*. However, the apparent mesh size obtained from *G*_0_ for K8/K18 without surfactant, vimentin and desmin is very low compared to the cubic grid model because *G*_0_ includes an additional contribution from stretched filament strands between contact points.

Table 1 compares the effect of surfactant on *G*_0_ for K8/K18, K8Δ/K18ΔT and vimentin. IF proteins without tail domains still form bona fide filaments [18], [31]. A higher concentration was chosen for vimentin because it consists of 32 instead of 16 molecules per cross-section as in K8/K18 filaments [22]. Without surfactant, *G*_0_ of K8Δ/K18ΔT is close to the result for the wild-type and also the vimentin network exhibits a *G*_0_ similar to that of the keratins. In the presence of TX-100, *G*_0_ of all samples decrease dramatically demonstrating that the phenomenon is not unique to K8/K18. This decrease is even more pronounced for K8Δ/K18ΔT and vimentin than for K8/K18. The torques measured by the rheometer to determine the moduli of these samples in oscillatory shear are close to the resolution limit of the device. The noise level of the rheometer characterized by the standard deviation of the torque signal during air measurements was 8.6±0.2 nNm. In this work, the torques of K8/K18 at *c*≥0.5 g/l with TX-100 were always above 12 nNm at *γ*≤0.2 using a 25 mm parallel plate geometry. The moduli of vimentin with TX-100 were measured using a 50 mm plate and a shear amplitude of *γ* = 0.15 to obtain torques above the noise level of the device (>10 nNm).

**Table 1:** Influence of Triton X-100 on *G*_0_ at *ω* = 0.5 rad/s.

IF protein	*c*	without surfactant	0.1% TX-100
	µM	Pa	Pa
K8/K18	9.8	3.9±0.8*	0.017±0.004*
K8Δ/K18ΔT	9.8	1.5	0.0025
vimentin	16.8	1.8	0.0022

*exp. errors calculated from st. dev. of at least three independent measurements.

### Non-linear viscoelasticity

**Figure 6:**
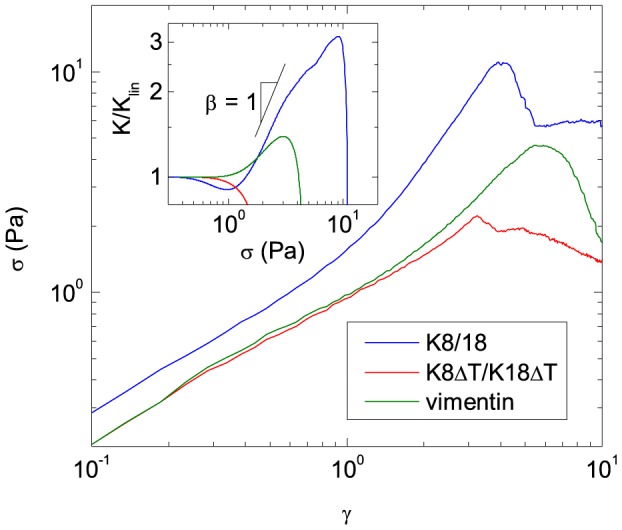
Shear stress *σ* versus deformation *γ* of different IF protein networks measured in steady shear. The concentration of K8/K18 and K8Δ/K18ΔT is 9.8 µM. The vimentin concentration is 16.8 µM. Measurements were done at a constant shear rate 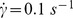. Inset: The differential modulus *K* derived from the stress-strain curves normalized by its value at small stresses *K*_lin_ as a function of *σ*.

The non-linear viscoelastic network properties were characterized by applying a constant strain rate of 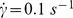 and measuring the resulting shear stress *σ*. Corresponding results for K8/K18, K8Δ/K18ΔT and vimentin are shown in Figure 6. All IF networks exhibit an increase of stress with increasing strain. When a critical strain *γ_max_* is exceeded, the network seems to rupture and the stress drops drastically. Strain stiffening is characterized by the increase in slope of the stress-strain curve. To quantify strain stiffening, we calculated the differential modulus *K* = d*σ*/d*γ* from the data in Figure 6. The differential modulus is constant in the linear elastic regime, increases in the case of strain stiffening, and drops at the point when the network apparently ruptures. The results presented in the inset of Figure 6 demonstrate that strain stiffening is much more pronounced for K8/K18 than for vimentin networks. This is evident from the lower slope of the *K*(*σ*)-curve and the lower maximum value of *K*. K8Δ/K18ΔT does not show strain stiffening although it assembles into uniform filaments [31]. The IF solutions with TX-100 exhibit *σ* values at least one order of magnitude lower than the corresponding surfactant-free networks and the stress response is approximately strain independent similar as for viscous fluids. Hence, the differential modulus is essentially zero.

## Discussion

### Network architecture

The good agreement between the mesh size determined from particle tracking and the theoretical value for a cubic network of individual filaments shows that the contour length *l*_c_ of the filaments between adjacent contact points is close to the mesh size *l*_c_≈*ξ*, which is reasonable since *l*_p_≥*ξ*. Bundling is not relevant for pure K8/K18 networks under the conditions investigated here. This is supported by the narrow distribution of MSDs, since bundling typically results in a broadening of the MSD distribution. Here, we find normalized standard deviations below 33% at *τ* = 1 s, which is similar to what has been reported for pure F-actin networks [32]. In contrast, bundling was observed for IF networks from keratin 5 and keratin 14 [17], [33] or from K8/K18 at pH 7.0 [14] or in the presence of salt [15], [34], [35].

The addition of TX-100 has no significant influence on the particle motion observed in microrheological experiments. Moreover, the surfactant has no effect on the rheological properties at high frequencies (Figure 2) and the visual impression of the K8/K18 filaments in electron micrographs (Figure 3A and B). Hence, the surfactant does not affect network parameters, such as filament diameter, persistence length, network heterogeneity or mesh size.

### IF-IF interactions at small deformations

Recently, we have proposed that thermodynamically unfavorable stretched filament conformations strongly contribute to the elastic network properties at low concentrations [15]. Therefore, *G*_0_ is orders of magnitude higher than expected for a network of semi-flexible chains especially at low protein concentrations. Furthermore, the dependence of *G*_0_ on concentration is weaker than predicted by statistical mechanical theories for networks of semi-flexible chains, since the contribution to *G*_0_ from stretched filaments between adjacent cross-links decreases with decreasing mesh size, i.e. increasing protein concentration. The scaling exponent for K8/K18 is *α* = 0.5±0.1, which is close to *α* = 0.58 derived for networks of chemically cross-linked flexible polymers swollen by a good solvent [16]. The proposed stretched filament strands can only exist if there is a strong attractive interaction among filaments at their contact points, otherwise the filaments would equilibrate to gain entropy.

The macroscopic mechanical properties of K8/K18 networks change dramatically in the presence of TX-100. The plateau modulus *G*_0_ decreases by orders of magnitude and is in the range expected for an equilibrated network. We can estimate *G*_0_ from the K8/K18 concentration in [g/l] by combining equation (1) and (3):
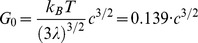
As shown in Figure 5A, the absolute values predicted by this simple estimate are close to the results from shear rheology in the presence of TX-100 and it seems that the additional contribution from stretched filament strands vanishes upon addition of the surfactant. The scaling exponent *α* = 1.9±0.2 is captured by various theoretical models for networks of semi-flexible or flexible polymers, which predict scaling exponents between 1.4 and 2.5 [8]–[11]. In addition, the concentration dependence and the absolute values of *G*_0_ show reasonable quantitative agreement with the simple estimate given in equation (4) indicating that only the cross-links contribute to the network elasticity. It should also be noted that the motion of tracer particles is determined by the mesh size of the network, but not by additional contributions to the free energy density, e.g. the stretched filament strands. Accordingly, there is a strong discrepancy between the *G*_0_ values from MPT and mechanical shear rheometry for untreated K8/K18 networks, but these values are in excellent agreement for the surfactant treated networks.

Figure 5B illustrates that the mesh size predicted from rheology for K8/K18 with TX-100 and actin [8], [36] is close to the mesh size assuming a cubic grid of filaments. This confirms that there is no significant additional free energy contribution from the filaments between cross-links. In contrast, data for IF networks without additional surfactant are well below the diagonal line, indicating an additional free energy contribution due to stretched filaments.

The plateau modulus of K8Δ/K18ΔT and vimentin without surfactant is close to that of the wild-type of K8/K18. In the presence of the surfactant, the plateau moduli of K8Δ/K18ΔT and vimentin are about one order of magnitude lower than for K8/K18 corresponding to a lower density of entanglements or cross-links. This might be attributed to a larger fraction of short filaments or dangling ends that do not contribute to the network elasticity. This hypothesis is further supported by the high value of *G″*/*G′*, which implies pronounced viscous losses during oscillatory shear of this networks. The estimated isoelectric points of K8ΔT and K18ΔT are 4.89 and 5.07. They are lower than the estimated isoelectric points of the wild-type with 5.26 for K8 and 5.11 for K18. Hence, the net negative charge of K8ΔT/K18ΔT at pH = 7.5 is higher than for the wildtype. Also vimentin exhibits strong electrostatic repulsion allowing strain stiffening only at high salt concentrations [7]. These repulsive interactions may also lead to a reduction of the cross-link density.

### Strain stiffening of IF networks

K8/K18 and vimentin show strain stiffening, which is characteristic for many biological gels [37]. Strain stiffening mainly originates from a strong non-linear increase of the entropic contribution to the free energy of the network when the filament strands between cross-links are highly stretched [20]. The glassy wormlike chain model (GWLC) [38] extends the classical wormlike chain model by introducing reversibly breakable cross-links, so-called sticky contacts, representing the attractive interactions at filament contact points. The model has been successfully used for a quantitative description of the mechanical properties of actin filament networks [21]. According to the GWLC model, the strength of these cross-links is characterized by the stickiness parameter *ε*. The change of the differential modulus with stress, characterized by the slope 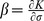, increases with increasing *ε* and approaches a limiting value of *β* = 3/2 for irreversible cross-links (*ε* →∞) [38].

**Figure 7:**
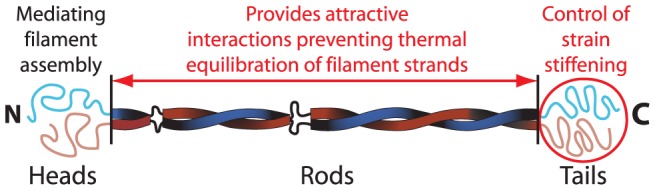
Schematic representation of the K8/K18 complex and the protein domains responsible for the network viscoelastity. Illustration adapted from [45].

**Figure 8:**
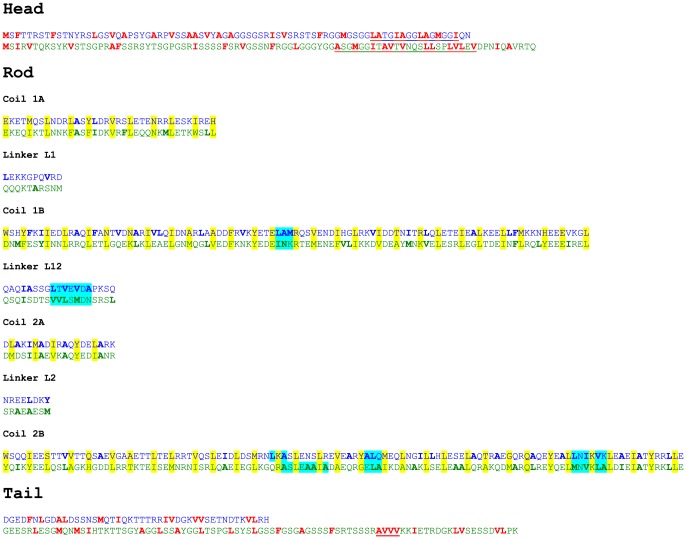
Amino acid sequence alignment of keratin assembly partners K18 (blue letters) and K8 (green letters). The order of the subdomains is as reported in Figure 1 of [46]. Hydrophobic amino acids in the non-α-helical head and tail domains are indicated in red [47]. Significant hydrophobic motifs in these domains are underlined. In the rod domain, the a- and d-heptad positions are highlighted in yellow; these amino acids are responsible for the formation of a coiled-coil dimer from two individual α-helices. Hydrophilic domains on the surface of a coiled-coil dimer, generated by amino acids positioned in the b-, c-, e-, f-, and g-positions of the heptad pattern, are highlighted in cyan.

The results presented in Figure 6 show that the bond strength characterized by the *ε*–value is weaker for vimentin than for K8/K18. This is presumably due to the strong electrostatic repulsion among filaments found for vimentin [7], which partly attenuates the attractive hydrophobic or van der Waals attractions. The value of *β* = 1 found for K8/K18 corresponds to a high, but finite *ε*–parameter. No strain stiffening is observed for K8Δ/K18ΔT. The strong influence of the tail domain on strain stiffening was also found for K14 [33], vimentin [12] and desmin [18] confirming that the protein sequence motifs providing the strong attraction at filament contact points are located in the tail with its high fraction of hydrophobic amino acids (Figure 7 and Figure 8).

### Surfactant mediated steric stabilization of IFs

The effect of added surfactant on the viscoelastic properties of IF networks can be rationalized by taking into account that the hydrophobic parts of the surfactant molecules adsorb onto hydrophobic regions of the keratin filaments, thus providing a steric stabilization. This prevents the filaments from approaching each other close enough to encounter strong attraction.

To bind or unbind a filament, an energy barrier characterized by the stickiness parameter *ε* has to be overcome and this determines the binding/unbinding kinetics [39]. The energy gain due to filament-filament bonds is so high for K8/K18 that two filaments always form bonds when they are close enough together. The unbinding rate approaches zero because of the high energy barrier *ε*. Therefore, filament strands between such contact points are not generally in thermal equilibrium and the entropically unfavorable stretched conformation of filaments between adjacent cross-links leads to an additional contribution to the free energy. The tail domain of IFs has no influence on the high plateau modulus values observed for K8/K18, vimentin [12], desmin [18] and keratin 14 [33]. Thus, the attractive hydrophobic interactions required to maintain the stretched filament strands between contact points, which are responsible for the high plateau moduli, are located in the central rod domain (Figure 7). Five particular hydrophobic amino acids clusters are found in the rod region of the K8 and K18 coiled-coil dimer in addition to one cluster each in the head domains close to the beginning of the rods (Figure 8). Despite the attractive interactions provided by these hydrophobic domains, filament bundling does not take place. The glutamic and aspartic acid groups distributed along the rod domain presumably prohibit the bundling. The electrostatic repulsive forces originating from these negatively charged groups prevents further contacts, which would lead to parallel bundling. K8/K18 filament only form bundles at higher ionic strengths at which the range of the electrostatic forces is strongly reduced [15], [40].

Within the framework of the GWLC model, the steric stabilization of filaments provided by TX-100 is expressed as a reduction of the *ε*-parameter. The corresponding increase in the binding/unbinding rates enables the filaments to attain their thermodynamically favorable conformation. Accordingly, the formation of sticky contacts or permanent cross-links is suppressed and equilibration of the stretched filaments between cross-links is enabled. Nevertheless, the surfactant does not change the number of contact points, which is determined by the protein filament length density and is well estimated by the simple cubic grid model (Figure 5A). As a result, the mesh size is unaffected by the addition of surfactant (Figure 3). Finally, the linear viscoelastic response of the networks in the high frequency regime is not affected by the surfactant, since it is determined by the stress relaxation of individual short filament strands and therefore, is independent of the interactions among filaments at their contact points (Figure 2).

Tailless mutants form networks with high *G*_0_ values (Table 1), but without showing strain stiffening (Figure 6) [12], [18], [33]. In this case, sticky contacts exist and are strong enough to prevent thermal equilibration of the filament strands between contact points, but not strong enough to withstand the high stresses occurring at large deformations.

## Conclusions

The viscoelastic properties of K8/K18 and vimentin networks without and with added non-ionic surfactant have been studied comprehensively using bulk mechanical rheometry (oscillatory shear and squeeze flow) and optical microrheology (DWS and MPT). The different methods yield consistent results and we can conclude:

1. The high *G*_0_ values at low concentration and the weak dependence of *G*_0_ on protein concentration, characteristic not only for keratin, but also for various other IF networks [5]–[7], emerge from a strong entropic contribution of stretched filament strands between filament contact points to the free energy of the networks [15]. This requires attractive interactions (>*k_B_T*) at these contact points, which are provided by the central rod domain of the protein.
2. Strain stiffening is another characteristic and physiologically relevant feature of IF networks. This requires stronger attractive forces at filament contact points. The protein sequences providing these attractions are located in the tail domain.

The formation of sticky contacts among filaments can be suppressed by adding non-ionic surfactant to the network. Then strain stiffening vanishes and the concentration dependence of *G*_0_ gets more pronounced. The corresponding scaling exponent is well captured by theoretical predictions for networks of semi-flexible or flexible polymers. For keratin K8/K18, the simple cubic grid model is a good approximation since the persistence length is on the order of the mesh size.

## Materials and Methods

### Protein preparation

Recombinant human wild-type and human tailless K8 and K18 proteins were prepared and purified as previously described [41]. K8 and K18 was mixed in a 1∶1 ratio and renatured by a stepwise dialysis from 8M Urea, 2 mM Tris-HCl (pH 9.0) and 1 mM DTT to 2 mM Tris-HCl (pH 9.0) and 1 mM DTT. Concentration of the individual proteins and the final concentration were determined by a Bradford assay (Bio-Rad) using bovine serum albumin as standard. The assembly was started by addition of an equal volume assembly buffer consisting of 18 mM Tris-HCl (pH 7.0) and 0–0.2% by weight of TX-100, resulting in 10 mM Tris-HCl (pH 7.4) and 0–0.1% TX-100. Vimentin was prepared recombinantly as described by Schopferer et al. [7]. The assembly was started by addition of 10× assembly buffer to obtain final concentrations of 25 mM Tris-HCl (pH 7.5), 160 mM NaCl and 0.1% TX-100. All samples were assembled in situ for 60 min at 20°C. All measurements were conducted at a temperature of 20°C.

### Electron microscopy

Transmission electron micrographs were taken after fixation with glutaraldehyde and negative staining with uranyl acetate according to Mücke et al. [4]. The filaments were assembled at a concentration of 0.4 g/l without surfactant and with 0.01% TX-100 and diluted to 0.2 g/l by addition of assembly buffer containing 0.1% glutaraldehyde.

### Shear rheology

The storage modulus *G′* and the loss modulus *G″* were measured in the frequency range between 10^−2^ and 10^2^ rad/s using a stress-controlled rheometer (Physica MCR 501; Anton Paar, Austria) with 25 mm and 50 mm plate-plate geometry. Results for samples without TX-100 are not influenced by plate diameter and gap width as shown in reference [15]. The critical TX-100 concentration, at which *G*_0_ drops, increases with decreasing gap width because the fraction of surfactant needed to saturate the external sample surface increases when the gap width is decreased. Above the critical TX-100 concentration, the viscoelastic moduli remain constant. Measurements were always conducted above the critical threshold, e.g. experiments with 25 mm plate at a gap width of 1.2 mm were conducted with 0.01% TX-100 and with 0.1% TX-100 at a gap width of 0.12 mm. Evaporation was minimized using a tempered hood and by maintaining a moist atmosphere. The frequency dependence of the moduli was obtained in the linear-viscoelastic regime, which was determined by preliminary amplitude sweeps. The non-linear rheological properties were characterized with 25 mm plate at a gap width of 0.12 mm by applying a steady strain rate 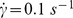 and measuring the resulting shear stress *σ*. The differential modulus *K* = d*σ*/d*γ* was calculated from the smoothed stress-strain curves.

### Squeeze flow

*G′* and *G″* data in the frequency range from 10 to 3·10^4^ rad/sec were obtained from oscillatory squeezing the sample at very low deformations using a piezo-driven axial vibrator as described in [7], [42].

### MPT

Green fluorescent, non-functionalized polystyrene tracer particles (Bangs Laboratories, USA) in dialysis buffer were added to the protein solution and mixed by vortexing before addition of the assembly buffer. After addition of the assembly buffer, both solutions were mixed and filled in a self-build sample chamber, which was sealed with an UV curing optical adhesive (NOA63, Norland Optical Adhesive, USA). The final particle concentration was 0.01%. The thermal motion of at least 50 particles was tracked at a temperature of 20°C and analyzed as described in [15].

### DWS

DWS measures the intensity correlation function (ICF) of the temporal fluctuations of light scattered by added tracer particles. The average MSD can be calculated from ICF for times between 10^−7^ and 10^1^ s [27]. Polystyrene particles (Invitrogen) with 1.3 µm diameter were coated with polyethylene glycol (PEG) following the swelling based approach of Kim et al. [43] using Pluronic F127 (BASF SE). The coated particles were washed with dialyses buffer and mixed with the protein solution by vortexing prior to assembly. The final particle concentration was 1%. Measurements were performed using 150 µl sample and glass cuvettes with a thickness of 1 mm (Hellma, Germany). The data was recorded and analyzed using the DWS ResearchLab (LS Instruments, Switzerland). The acquisition time was set to 270 s and data analysis was done as described in [44].

## Supporting Information

Figure S1 ***G′*** **(closed symbols) and** ***G″*** **(open symbols) for K8/K18 networks without surfactant (red) and with 0.01% TX-100 (blue) measured by MPT (circles) and DWS (diamonds).** The K8/K18 concentration is 1.0 g/l.
(EPS)
