# Correction: Attractive Interactions among Intermediate Filaments Determine Network Mechanics In Vitro

**DOI:** 10.1371/journal.pone.0109450

**Published:** 2014-09-29

**Authors:** The PLOS ONE Staff

There is an error in Equation 2. Please see the corrected Equation 2 here.
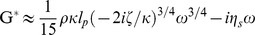



The equation numbering is missing in the published article. Please see the equations and their associated numbers here.
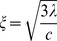
(1)

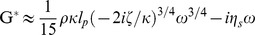


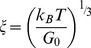
(3)

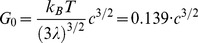
(4)

